# Acral papular mucinosis: a new case of this rare entity*

**DOI:** 10.1590/abd1806-4841.20164804

**Published:** 2016

**Authors:** María Encarnación Gómez Sánchez, Fernando de Manueles Marcos, Maria Luisa Martínez Martínez, Roberto Vera Berón, Jose Manuel Azaña Défez

**Affiliations:** 1 Hospital General de Villarrobledo – Villarrobledo-Albacete, Spain; 2 Complejo Universitario Hospitalario de Albacete – Castilla la Mancha, Spain

**Keywords:** Mucinoses, Scleromyxedema, Therapeutics

## Abstract

Acral persistent papular mucinosis (APPM) is a rare subtype of localized lichen myxedematosus. It consists of small papules localized exclusively on the back of the hands, wrists and extensor aspects of distal forearms with no other clinical or laboratory manifestations. The lesions tend to persist and may increase slowly in number. Histologically, hematoxylin-eosin and Alcian blue staining demonstrate mucin accumulation in the upper reticular dermis with separation of collagen fibers as a result of hyaluronic acid deposition. Treatment is rarely necessary due to the absence of symptoms. We present a 27-year-old healthy woman with asymptomatic papules on her upper extremities, which adequately meet clinical and pathological criteria of acral papular mucinosis.

## INTRODUCTION

Cutaneous mucinosis is a group of disorders charaterized by an accumulation of mucin or glycosaminoglycan in the skin and its annexes.^3^ Acral persistent papular mucinosis (APPM) is a rare subtype of localized lichen myxedematosus. To date, there have been only 34 reported cases of this entity that strictly fulfilled the diagnostic criteria proposed by Rongioletti and Rebora in 2001.^1^

We present a 27-year-old healthy woman with asymptomatic papules on her upper extremities, which adequately meet clinical and pathological criteria for acral persistent papular mucinosis.

## CASE REPORT

Our patient complained of persistent, asymptomatic and symmetrical skin lesions on the hands and arms, which evolved over a period of one year. She had no history of medical problems and reported no use of oral or topical medications. She reported no previous injuries or trauma to the affected sites and we could establish no relationship with sun exposure. No other family member had been affected.

**Figure 1:**
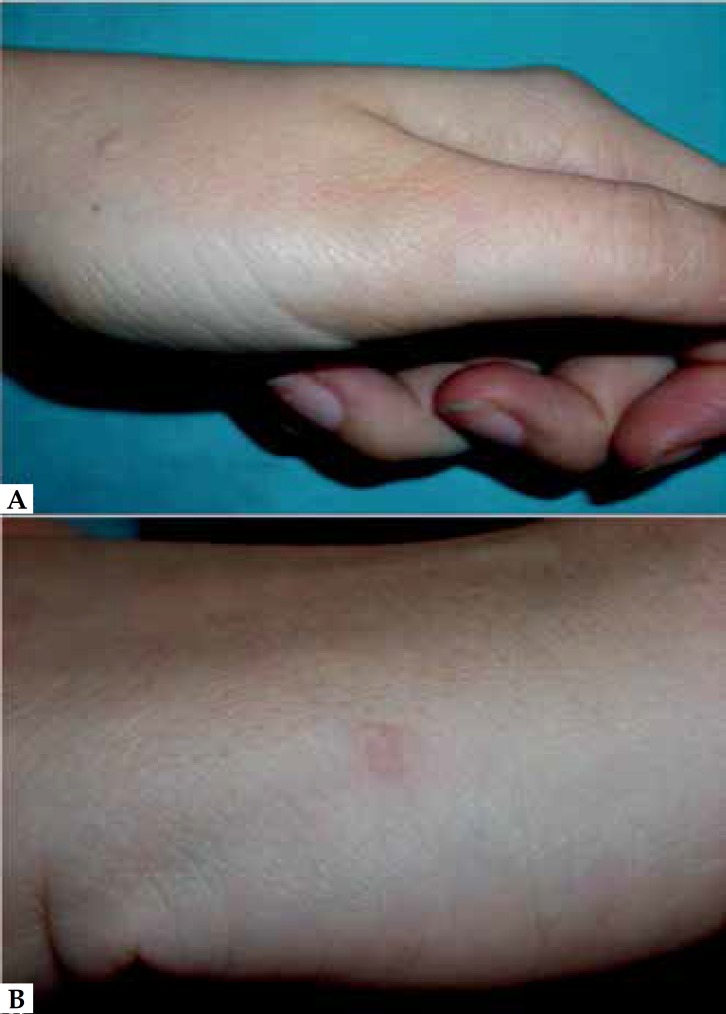
Multiple flesh colored millimeter-sized papules distributed on the back of the hand **(A)** and on the distal forearm **(B)**

**Figure 2:**
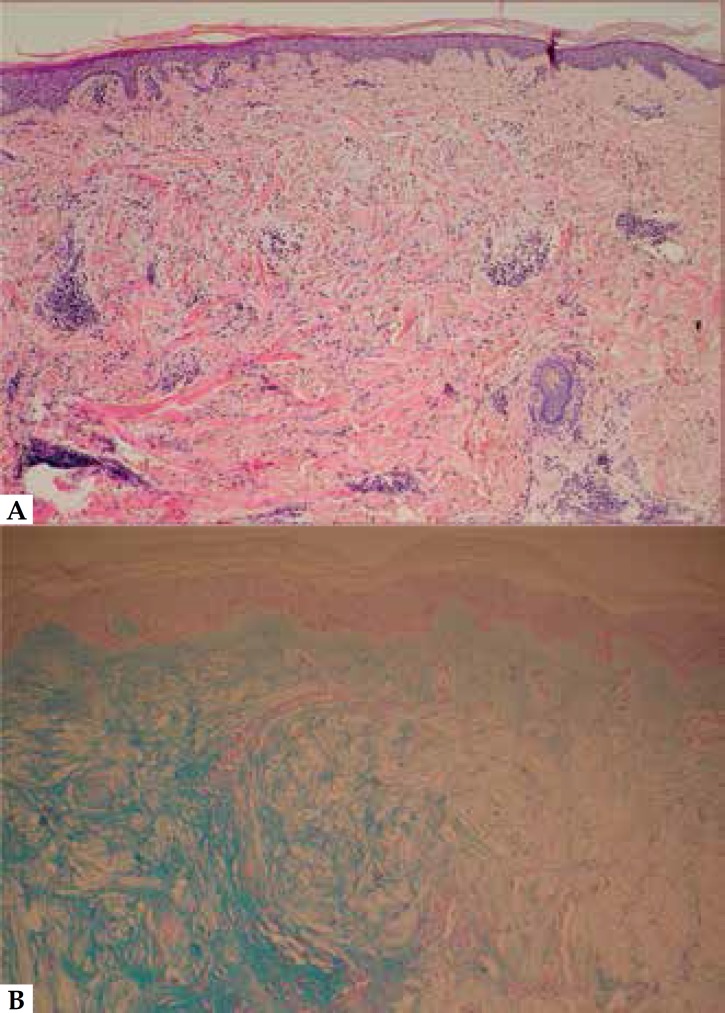
**(A)** Normal epidermis and a focal lighter area in the upper and mid reticular dermis due to a separation of collagen fibers by mucin accumulation (HE stain 60x). **(B)** The circumscribed area is positively stained with colloidal iron, indicating mucin accumulation (Colloidal iron staining 100x).

Physical examination revealed approximately 8 small (3-5 mm) firm round skin-colored papules located exclusively and symmetrically on the dorsum of the hands and wrists (Figure 1). We identified no lesions on the surrounding skin. A 4-mm punch biopsy specimen from a papule showed epidermis without alterations and a distinguished localized deposit of mucin in the papillary and upper reticular dermis that stained positively with colloidal iron staining (Figure 2). There was not an elevated fibroblast count. Results from laboratory studies were normal, including thyroid function tests and serum protein electrophoresis. The patient was diagnosed with acral persistent papular mucinosis. Due to scarce symptomatology, she refused to be treated at that time.

## DISCUSSION

Cutaneous mucinoses are a heterogeneous group of disorders in which an abnormal amount of mucin is accumulated in the skin and their etiopathogenesis is still unknown.^1^ The condition is traditionally divided into two groups: primary mucinosis – in which mucin deposits are the main histologic feature manifested through cutaneous signs; or secondary mucinosis – additional and casual findings in the biopsy specimen of other diseases. The latter group includes: certain endocrinopathies (especially thyroid diseases); toxic diseases (toxic oil syndrome); Eosinophilia-Myalgia Syndrome, nephrogenic fibrosing dermopathy; and diffuse connective tissue diseases (such as lupus erythematosus, and cancer).^1-3^

The latest classification of dermal mucinosis was established by Rongioletti and Rebora in 2001.^4,5^ They differentiated two main groups of dermal mucinosis: the generalized form or scleromyxedema, which has a constant association with systemic disorders (like paraproteinemia) or less frequently with hematologic malignancies; and the localized form, which does not have a systemic involvement, also called lichen myxedematosus (LM). Cases not meeting the criteria for scleromyxedema or localized form are classified as atypical.^1,2.4-9^ Acral persistent papular mucinosis was first reported by Rongioletti *et al.* in 1986^2,4-8^ as one of the five subtypes of lichen myxedematosus. By definition, the small papules are localized exclusively on the back of the hands, wrists and extensor aspects of distal forearms with no other clinical or laboratory manifestations.^7^ The lesions tend to persist and may increase slowly in number.^4,8^ Histologically, hematoxylin-eosin and Alcian blue staining demonstrate mucin accumulation in the upper reticular dermis with separation of collagen fibers as a result of hyaluronic acid deposition. Mucin accumulation may cause thinning of the epidermis.^6^ To date, 34 cases have been reported, four of whom presented with a past history of malignant tumors.^6,8^ Luo *et al.*^8^ summarized the reported cases and confirmed a female predominance. The main age at onset was 42.9 ± 15.9. The possible association with malignancies has not been clarified yet. Treatment is rarely necessary due to the absence of symptoms. Topical corticosteroids, tacrolimus and pimecrolimus have been used with some success.^6,7,8^

We presented another case of this rare type of acral mucinosis. It is important to emphazise that it is approriate excluded other systemic or secondary forms the disease in case of finding any skin accumulation of mucin.
